# Hypersomnolence in focus: a white paper of the 6th Think Tank World Sleep Forum

**DOI:** 10.1016/j.sleep.2025.106607

**Published:** 2025-06-06

**Authors:** Alessandro Silvani, Claudio Bassetti, Matteo Bradicich, Richard Dodel, Luigi Ferini Strambi, Göran Hajak, Jan Hedner, Raphael Heinzer, Sogol Javaheri, Poul Jennum, Ulf Kallweit, Ramin Khatami, Gert Jan Lammers, Jean-Louis Pepin, Fabio Pizza, David T. Plante, Giuseppe Plazzi, Winfried Randerath, Joan Santamaria, Sophia Schiza, Tina Sundelin, Wendy Troxel, Mauro Manconi

**Affiliations:** a Dept of Biomedical and Neuromotor Sciences, University of Bologna, Bologna, Italy; b Dept of Neurology, Inselspital, University of Bern, Bern, Switzerland; c Dept of Pulmonology, University Hospital Zurich, Zurich, Switzerland; d Dept of Geriatric Medicine, University of Duisburg-Essen, Germany; e Sleep Disorders Center, Vita-Salute San Raffaele University, Milan, Italy; f Munich Psychiatry Outpatient Clinic, Munich, Germany; g University of Regensburg, Germany; h Dept of Internal Medicine and Clinical Nutrition, Institute of Medicine, Sahlgranska Academy, University of Gothenburg, Gothenburg, Sweden; i Centre for Investigation and Research in Sleep (CIRS), University Hospital of Lausanne, Lausanne, Switzerland; j Division of Sleep and Circadian Disorders, Brigham and Women’s Hospital and Harvard Medical School, Boston, MA, USA; k Danish Center for Sleep Medicine, Dept of Clinical Neurophysiology, University of Copenhagen and Danish Technical University, Copenhagen, Denmark; l Professorship for Narcolepsy and Hypersomnolence Research, Faculty of Health, University Witten/Herdecke, Witten, Germany; m Centre of Sleep Medicine and Sleep Research, Klinik Barmelweid AG, Barmelweid, Switzerland; n Sleep Wake Centre, Stichting Epilepsie Instellingen Nederland, Heemstede, the Netherlands; o Department of Neurology, Leiden University Medical Centre, Leiden, the Netherlands; p University Grenoble Alpes, INSERM U1300, CHU Grenoble Alpes, HP2 Laboratory, Grenoble, France; q IRCCS, Istituto delle Scienze Neurologiche di Bologna, Bologna, Italy; r Dept of Psychiatry, University of Wisconsin-Madison, Madison, WI, USA; s Clinic of Pneumology and Allergology, Center for Sleep Medicine and Respiratory Care, Bethanien Hospital, Solingen, Germany; t Sleep Unit, Dept of Neurology, Hospital Clínic of Barcelona, Barcelona, Spain; u Dept of Respiratory and Sleep Medicine, School of Medicine, University of Crete, Greece; v Dept of Psychology, Stockholm University, Stockholm, Sweden; w Dept of Clinical Neuroscience, Karolinska Institutet, Solna, Sweden; x Division of Social and Economic Wellbeing, RAND, Pittsburgh, PA, USA; y Sleep Medicine Unit, Neurocenter of Italian Switzerland, Civic Hospital of Lugano, Ente Ospedaliero Cantonale (EOC), Lugano, Switzerland; z Faculty of Biomedical Sciences, Università della Svizzera Italiana, Lugano, Switzerland

**Keywords:** Excessive daytime sleepiness, Excessive need for sleep, Hypersomnia, Hypersomnolence, Obstructive sleep apnea, Mechanisms, Quality of life

## Abstract

An international expert group (European Sleep Foundation Think Tank) convened in 2022 to discuss the state of the evidence in the domain of hypersomnolence. The expert group considered the current state of knowledge based on the most relevant recent publications, discussed the current challenges in the field and identified future priorities. The purpose of this white paper is to summarize the definition, diagnosis, and pathophysiology of hypersomnolence, the epidemiology, phenotype, and management of hypersomnolence in obstructive sleep apnea and in neurological and psychiatric disorders, and the impact of hypersomnolence on daily activities, workability and health-related quality of life. The key results of the discussion were that: a) hypersomnolence is both prevalent and heterogeneous in its manifestations in a wide variety of pathological conditions encompassing obstructive sleep apnea and neurological and psychiatric disorders; and b) while multiple pathophysiological pathways are potentially involved in hypersomnolence, knowledge of the specific causal factors in individual patients remains undefined, and the specific factors responsible for excessive daytime sleepiness vs. excessive need for sleep remain largely unclear. The clinical implications of these results are the occurrence of important limitations to the development of personalized approaches to diagnosis, prognosis, and management of hypersomnolence, which is essential considering the high societal and personal costs of hypersomnolence, and its substantial adverse impact on quality of life. Research priorities should address these limitations with improved quantification of hypersomnolence and with an evidence base on the costs and benefit of hypersomnolence management in patients with respiratory, neurologic, and psychiatric disorders.

## 1. Introduction

The word “hypersomnolence” is an augmentation of “somnolence”, which may be considered a synonym of “sleepiness” and indicates a general physiological tendency to shift from wakefulness to sleep. Viewing sleep as an instinct, somnolence represents the *appetitive* phase, which precedes the *consummatory* phase of sleep. The prefix “hyper” in “hypersomnolence” signals the border between normal physiology and pathological symptoms, when somnolence becomes intense, prolonged, or occurs at undesired, unusual times outside the physiological permissive gates of sleep and thereby interferes with quality of life. When hypersomnolence translates into excessive sleep time (including ≥10 h of sleep per 24 h [1], and usually more than 11 h per day [2], it is more appropriately defined as “hypersomnia”.

This white paper summarizes the most important recent research and clinical findings concerning different aspects of hypersomnolence, starting from definitions and pathophysiology, progressing to epidemiology, diagnosis, and management of hypersomnolence in obstructive sleep apnea (OSA) and in neurological and psychiatric disorders, and concluding with the impact of hypersomnolence on daily activities, work productivity, and health-related quality of life. The main aim of the panel discussion was to identify research priorities in the field for the next decade.

## 2. Methods

This white paper summarizes the results of a collaborative effort by a group of international experts in relevant fields of interest, who convened for a 3-day workshop (Think Tank 2022) organized by the European Sleep Foundation. This paper follows a standardized approach used in previous Think Tank editions [3]. International experts, with expertise in sleep research and clinical care, neurology, psychiatry, physiology, and public health, participated in an in-person meeting, held from October 14 to October 16, 2022 in Baveno, Italy. This expertise, which spanned nine European countries and the U.S.A., included consideration of different experiences and healthcare systems. Three major topic areas were addressed including (1) definitions and diagnosis of hypersomnolence; (2) epidemiology, phenotypes, and management of hypersomnolence in OSA, neurological disorders, and psychiatric disorders; and (3) impact of hypersomnolence on daily activities, work productivity, and quality of life.

The participating experts were divided into five subgroups (diagnosis and definitions, pathophysiology, OSA, neurological disorders, psychiatric disorders, and quality of life). Each subgroup presented a draft document summarizing their opinion on the most important current data and research priorities in each field [3]. Each subgroup was tasked to select by expert opinion and discuss the most relevant publications in the last 5 years and the most relevant ongoing studies, hot topics, and topics recently addressed in international congresses. Finally, each subgroup identified and discussed urgent and long-term questions to address and a research agenda with expected results. The initial documents were then integrated into a manuscript by the task force coordinators, which was revised and updated by each task force participant. The subgroups worked together both during specific sessions of the in-presence meeting and with e-mail exchanges for the manuscript draft preparation and revision. The strategy adopted during the in-presence meeting and subsequent e-mail and document draft exchanges among the participating experts was to continue discussion until a unanimous consensus was reached or, if not possible, to explicitly report discrepant opinions of panel members. The latter option was not eventually needed because the group managed to reach consensus on all issues reported in this white paper.

## 3. What is hypersomnolence and how to assess it

Hypersomnolence is the umbrella term for manifestations of abnormally increased daytime sleepiness. A distinction can be made between complaints in the context of an inability to stay awake during the day without an increased duration of sleep time over 24 h, versus complaints associated with or despite a (greatly) increased duration of nighttime sleep. This second phenotype is not necessarily accompanied by actual unintended daytime sleep. The inability to stay awake during the day is called “excessive daytime sleepiness” (EDS), and the increased need for sleep “hypersomnia” or “excessive need for sleep” (ENS).

It is important to realize that EDS/ENS is not just characterized by the complaints of increased daytime sleepiness and unintended sleep, but is a multifaceted problem as reflected in recently proposed definitions (Table 1). For instance, impaired sustained attention contributes greatly to disease burden because it leads to performance impairments [1]. Expressions of EDS/ENS may also include other manifestations such as memory complaints, emotional changes such as irritability, as well as headache complaints or hyperactivity, particularly in children.

**Fig. 1.**
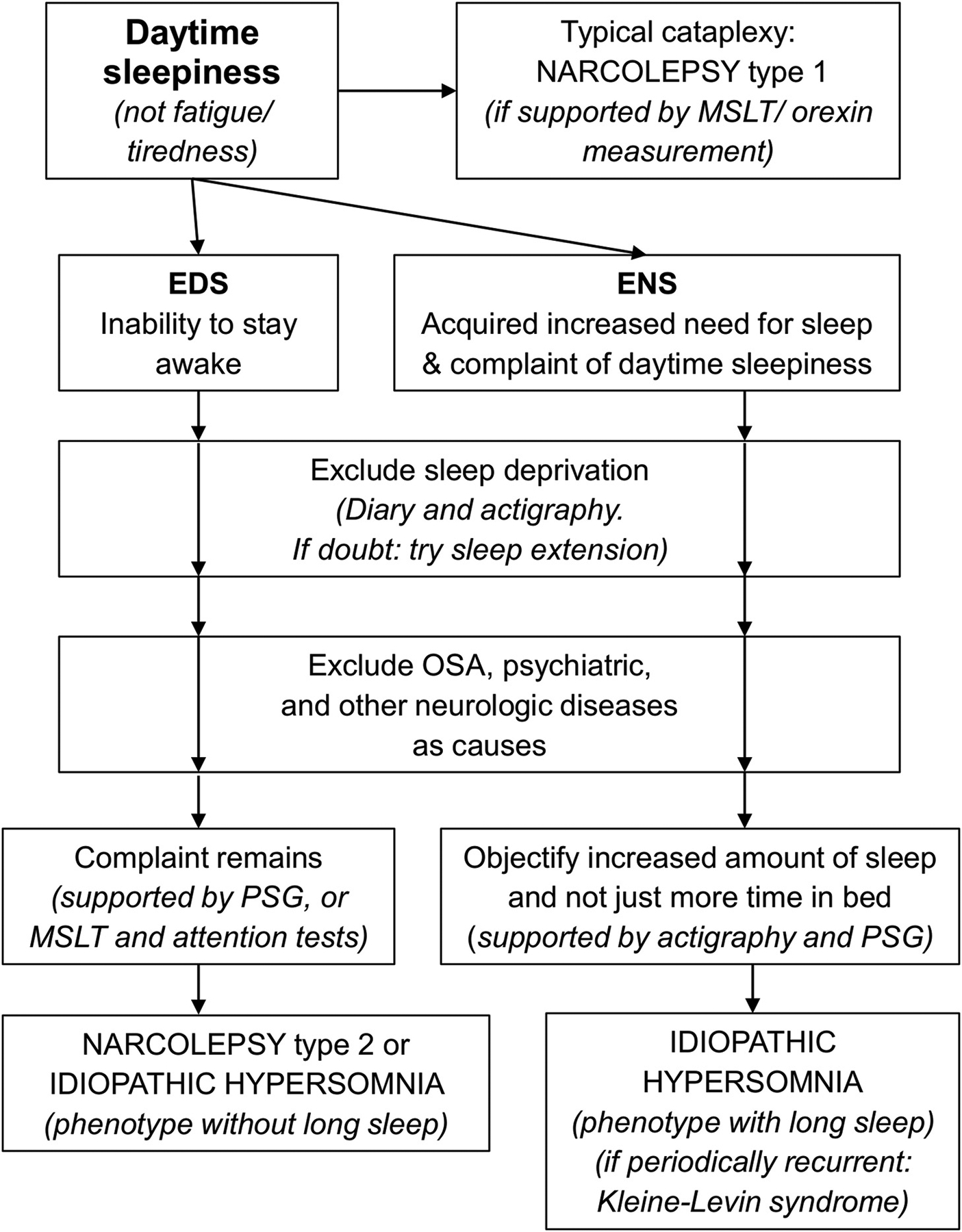
Proposed diagnostic workup for central disorders of hypersomnolence The diagram shows the hierarchy of clinical reasoning and investigations that may be done to arrive at a diagnosis of one of the central hypersomnolence disorders. The occurrence of typical cataplexy is at the top of the hierarchy: its presence indicates a diagnosis of narcolepsy type 1, even with comorbid sleep apnea or chronic sleep deprivation. Absent typical cataplexy, two phenotypes are possible: inability to stay awake during the day with no increased amount of daytime sleep (excessive daytime sleepiness, EDS, left column), or an increased amount of sleep and, despite that, complaints of excessive daytime sleepiness (excessive need for sleep, ENS, right column). In these situations, chronic sleep deprivation must first be ruled out as the cause, then obstructive sleep apnea (OSA, if hypersomnolence disappears after OSA treatment) and other neurological and psychiatric diseases. If these alternatives are ruled out and diagnostic criteria are fulfilled, the diagnoses of narcolepsy type 2 or idiopathic hypersomnia may be considered. PSG, polysomnography, possibly performed for 24 h. MSLT, multiple sleep latency test.

On the other hand, the increased need for sleep in hypersomnia should not be confused with the mere desire for sleep, which is expressed as prolonged time in bed and may occur in some depressed patients. Natural long sleepers may also report ENS (9–10 h of sleep per night), but they usually feel refreshed and do not complain of EDS when they wake up from extended sleep time. Fig. 1 illustrates a suggested diagnostic approach for people with a complaint of excessive daytime sleepiness.

EDS/ENS may be accompanied by fatigue (possibly referred to as exhaustion, weariness, tiredness), which is the sensation/feeling of extreme tiredness or exhaustion, a lack of energy that usually interferes with the person’s daily life. Fatigue is a primarily subjective complaint that is not necessarily associated with increased sleepiness. Particularly ENS often involves concomitant sleep inertia complaints. Sleep inertia is defined as the difficulty in waking up and achieving complete wakefulness at the end of a sleep period, potentially accompanied by confusion, disorientation, irritability, amnesia, and poor motor coordination or even ataxia [1,4]. Sleep “drunkenness” or “hang over” is considered as a severe manifestation of this phenomenon [5].

The distinction between sleepiness and excessive sleepiness is not always clear-cut, nor is the distinction between the physiological need for sleep and a pathological increased need for sleep. In general, with a naturally high sleep requirement, there will be no complaint during the day when this need is met. Also, a daytime sleep requirement is only excessive if the complaint persists on a daily or near-daily basis despite sleep extension [6]. One sleep disorder typically characterized by ENS is idiopathic hypersomnia with long sleep duration. Interestingly, although the prevalence of idiopathic hypersomnia has been estimated at 0.002–0.010 %, 3.5 % of older adults report sleeping ≥10 h per 24 h [7] and 1.6 % of adults report sleeping ≥9 h per 24 h with distress or impairment [8]. An acquired increase in sleep need >9 h with accompanying complaints of EDS/ENS despite extending the duration of nocturnal sleep should also be carefully considered for treatment.

EDS/ENS may also present clinically meaningful differences based on age. Young children may abruptly change sleeping habits with anticipation of nocturnal bedtime coupled with reappearance of daytime napping behavior, unintended daytime napping, or prominent behavioral abnormalities [9]. In somewhat older children/adolescents there may be behavioral/emotional changes, including irritability, aggressive expressions or emotional lability, and expressions very similar to those of attention-deficit/hyperactivity disorder (ADHD) or autism spectrum disorders (ASD). Especially inattention and hyperactivity are common in children [10] and may overlap with ADHD in clinical populations with properly defined narcolepsy [11].

The specific features of hypersomnolence in children and teenagers are summarized in Box 1. The onset of central disorders of hypersomnolence is rarely observed in old age. Nevertheless, in the elderly, the manifestations of hypersomnolence may also be different, for example, if there is a more sedentary lifestyle that facilitates falling asleep. Moreover, in general population cohorts, the complaint of EDS (as assessed with the Epworth Sleepiness Scale) was reported to decrease with age, being 2.9 and 2.8 times higher (95 % confidence intervals: 1.6–5.4 and 1.5–5.1) in subjects 40–49 and 50–59 years old, respectively, than in subjects 70–80 years old [12].

Objective measurement of hypersomnolence can only be done reliably in people who are not sleep-deprived. In clinical practice, however, this may be hard to determine with certainty, both because there are interindividual differences in sleep need and because there is no worldwide consensus on how to quantify it. The isolated use of a sleep diary is insufficient due to recall bias and inherent challenges of self-reporting behavior associated with altered consciousness (i.e., sleep). A good patient history and the use of actigraphy are considered indispensable, although this is not completely reliable and is based largely on expert opinion for now [1].

The Multiple Sleep Latency Test (MSLT, [13,14]) has long been considered the gold standard for quantifying EDS, and therefore the most important diagnostic test measuring the ability or propensity to fall asleep in a sleep-inviting setting. The outcome measure is the average sleep latency in minutes over usually 5 sessions in which people lie down in a dark quiet room and try to fall asleep. The MSLT is very sensitive to sleep deprivation and sleep extension, which makes it essential to only apply the test when sleep deprivation has been excluded, and its sensitivity and specificity in determining hypersomnolence have been criticized [15]. It is useful for assessing EDS but much less so for ENS. It should be stressed that MSLT primarily identifies indications of EDS but not always ENS and not at all fatigue or sleep inertia.

The Maintenance of Wakefulness Test (MWT) is designed to quantify the severity of EDS/ENS by measuring the patient’s ability to stay awake in sedentary circumstances, but is also affected by many physiological, psychological, and operational variables [13]. The applicability of the MWT to measure treatment effects in central disorders of hypersomnolence has been recently confirmed, but age-adjusted norm values are lacking, and its combination with subjective measures is required for a more complete evaluation of EDS [16]. The lower normal limit was estimated at 19.4 min based on the mean sleep latency to the first epoch of sustained sleep of healthy subjects allowed a maximum trial duration of 40 min [17].

Unfortunately, there is no universally accepted and well-validated test for ENS. Several polysomnography protocols, sometimes including MSLT, have been proposed to measure prolonged sleep duration [18, 19], but they are not standardized and they are difficult to apply due to being complex, lengthy, and expensive. Table 2 summarizes differences between MSLT and MWT and their relative appropriateness in different conditions.

### 3.1. Research priorities

There is a need for more research leading to a consensus on how to objectify and quantify hypersomnolence, including ENS, also addressing the role of actigraphy, especially in subjects who are acutely or chronically sleep-deprived. Ultimately, development and validation of sleep study protocols that distinguish EDS in narcolepsy versus long sleep need in idiopathic hypersomnia are required to diagnose different hypersomnia conditions more accurately and precisely. In addition, there is currently no accepted objective test for identifying and quantifying sleep inertia. It is possible that vigilance tests could identify and quantify this phenomenon [20], but further validation is needed.

## 4. Pathophysiology of hypersomnolence

**Fig. 2.**
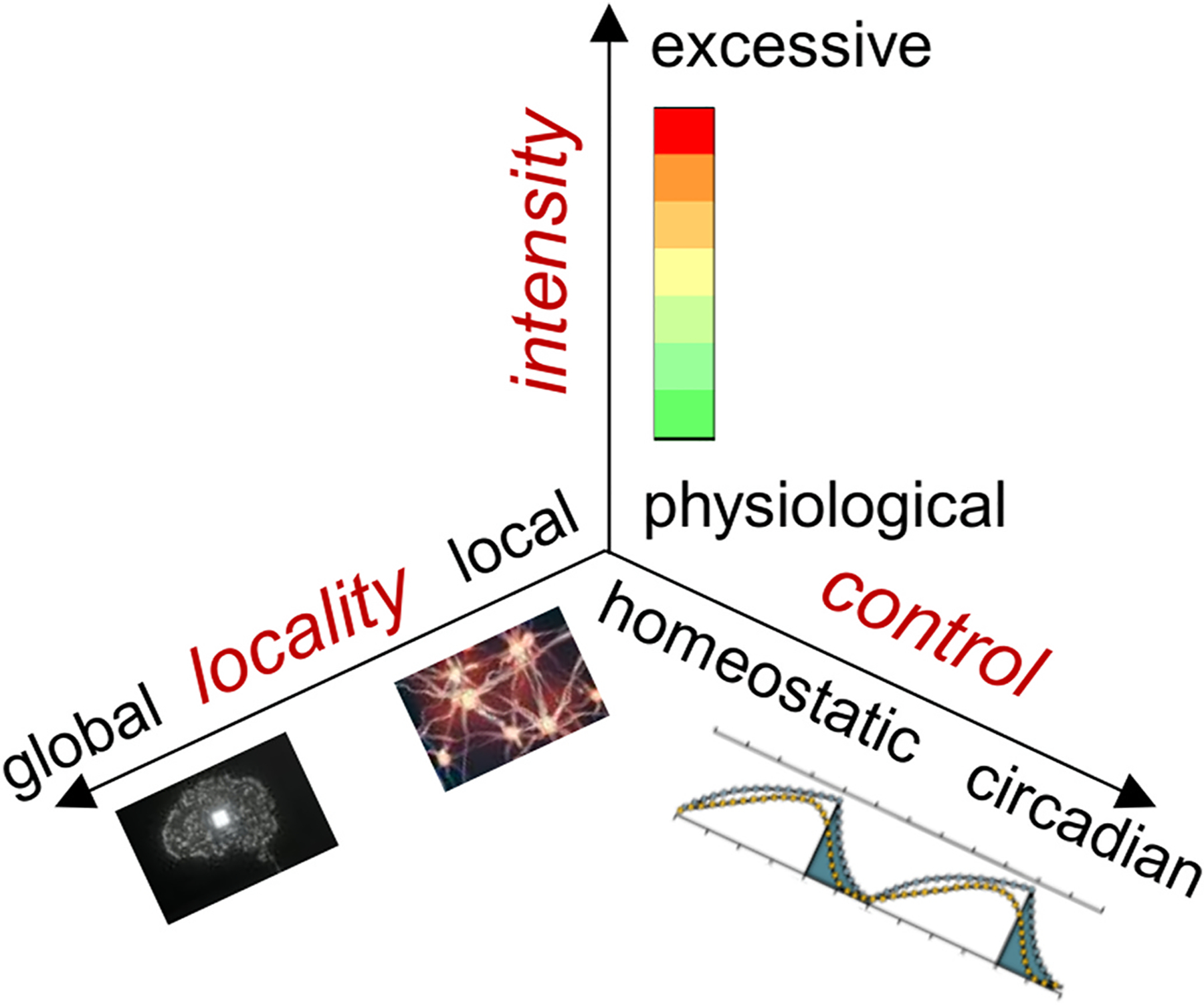
Intensity, locality and control in hypersomnolence pathophysiology The figure summarizes the proposed categorization of hypersomnolence pathophysiological mechanisms along three main axes: intensity (ranging from physiological through excessive), locality (ranging from local through global neural circuits), and control (ranging from predominantly homeostatic to predominantly circadian). The circadian/homeostatic control picture is taken from Ref. [159] (CC-BY license).

Reports on the pathophysiology of hypersomnolence are rapidly accumulating, and so is new knowledge on sleep physiology in relation to hypersomnolence. The available evidence on hypersomnolence pathophysiology may be conceptualized along three axes (Fig. 2): 1) physiological (adaptive) vs. pathological (excessive) sleepiness; 2) local vs. global brain neural circuits; 3) homeostatic vs. circadian regulation. Potential mechanisms include increased sleep drive or need, insufficient arousal or maintenance of wakefulness, and dysregulated circadian drive. Susceptibility factors include age, genetic and epigenetic makeup [21–23], and environmental factors [24]. Potential modulators include baroreceptor and autonomic function [25], visceral afferent activity [26] and inflammation [27,28].

The relevant brain structures may range from local sleep-promoting microcircuits to global wake-promoting and sleep-promoting circuits. Local sleep-promoting microcircuits may link sleep with synaptic plasticity [29]. Global wake-promoting and sleep-promoting circuits include the circadian master clock circuitry centered in the suprachiasmatic nuclei as well as mostly mutually inhibitory (“flip-flop”) circuits controlling wake-sleep transitions [30]. These circuits are in the brainstem, overlapping with the central autonomic network [31], hypothalamus, and basal forebrain, as well as rostrally in the paramedian thalamus [32], striatum, ventral tegmental area, nucleus accumbens, and cortex [33]. The neurochemistry of hypersomnolence may involve both sedative neurotransmitters like GABA, adenosine and melatonin, and alerting/wake-promoting neurotransmitters such as noradrenaline, dopamine, serotonin (mainly through 5HT_2c_ receptors), histamine, and orexin.

Plausible molecular mechanisms of hypersomnolence include the accumulation of adenosine due to glial-neuronal interactions [34] and the phosphorylation of sleep-need-index phosphoproteins by the Sik3 protein kinase [35], which are both associated with sleep drive. Recent data also support the role of Ca^2+^/calmodulin-dependent protein kinase II as a core sleep regulator in mammals [36].

EDS has a limited trait heritability and shared genetic determinants with psychiatric diseases and with orexin signaling [23]. Epigenetic wake-sleep control through miRNA has been demonstrated for orexin expression [37] and might also be involved in hypersomnolence [38].

**Fig. 3.**
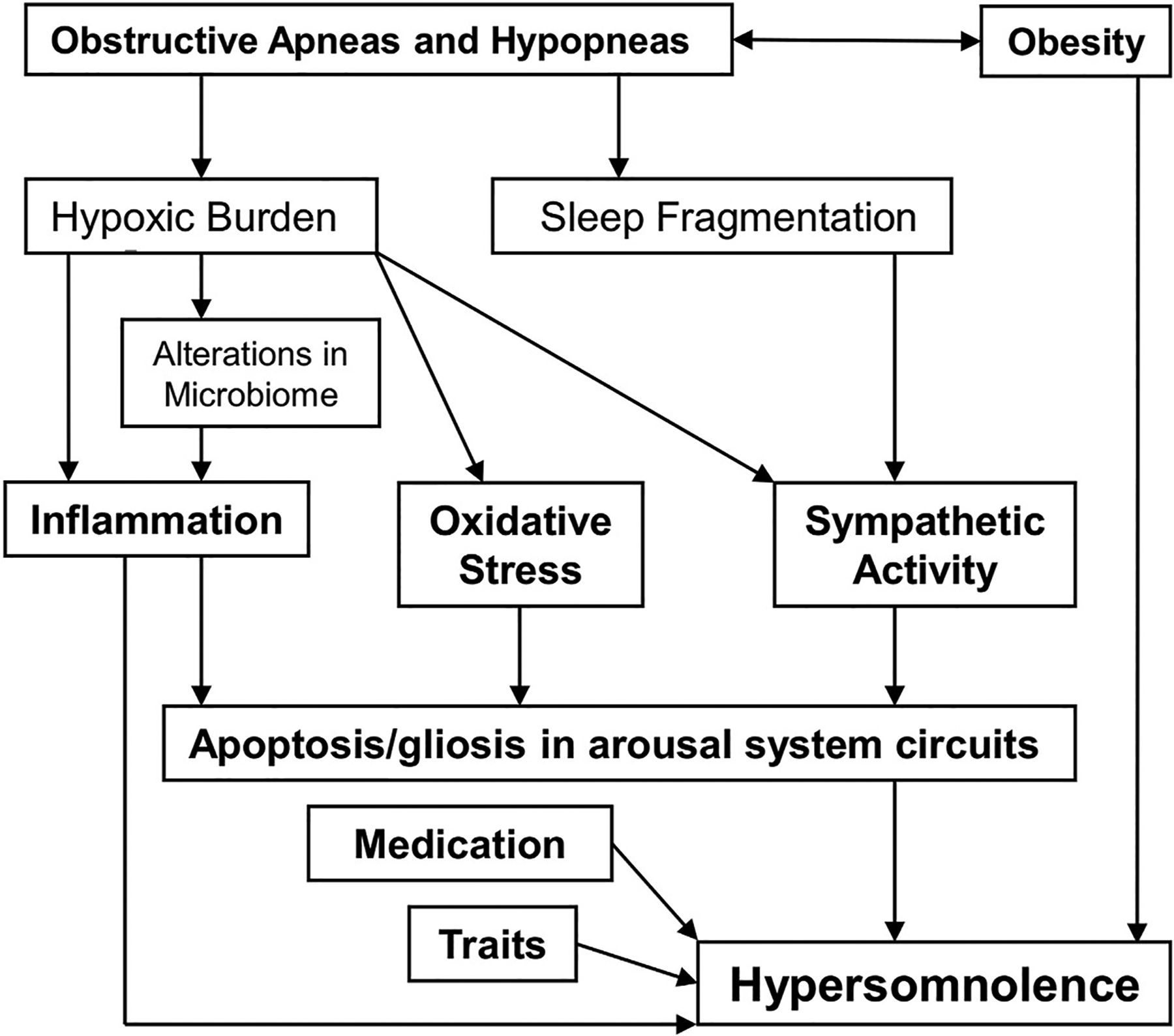
Specific mechanisms of excessive daytime sleepiness in obstructive sleep apnea. Figure adapted from Ref. [66], with permission.

The main mechanisms underlying hypersomnolence specifically in OSA (see section 5) are sleep fragmentation, hypoxic burden, and inflammation, which all may influence cardiovascular morbidity and mortality due to sympathetic activation, oxidative stress, and endothelial dysfunction (Fig. 3). This raises the hypothesis of common mechanisms inducing both hypersomnolence and cardiovascular complications in OSA. Genomics and proteomics are promising approaches for future research on the pathogenesis of hypersomnolence in OSA.

### 4.1. Research priorities

Much critical detail is still missing to develop coherent causal mechanistic models of the pathophysiology of hypersomnolence. Urgent questions concern the pathophysiological roles of autonomic and inflammatory alterations, the shared pathophysiology between EDS and ENS, and the pathophysiological specificity of objective vs. subjective hypersomnolence while considering the external validity of objective tests [39]. The research agenda should include the development of an integrative model of hypersomnolence, inflammation, and autonomic dysfunction, as well as the development of consensus criteria for valid animal models of hypersomnolence. In perspective, this should lead to mapping molecular links between sleepiness, hypersomnolence, and synaptic homeostasis and to identifying hypersomnolence biomarkers based on pathophysiology. The expected result is the development of therapeutics (pharmacologic and behavioral) or devices to treat hypersomnolence and prevent its adverse effects, including risk of cardiovascular disease, with appropriate attention to ethical considerations in collection of data, patient consent, and appropriate use of any genetic test information [40].

## 5. Hypersomnolence in OSA

### 5.1. Epidemiology and phenotypes

Hypersomnolence, especially EDS, is the main symptom of OSA, not only due to its high impact on daytime performance and ability to drive [41–45], but also due to its possible association with depression [46], and ultimately on lifestyle behaviors (e.g., physical activity, food intake) and work productivity. However, recent epidemiological data have shown that about half of patients with OSA present only with mild symptoms of hypersomnolence or even with insomnia complaints [47–51]. OSA diversity and complexity is vast, presenting sex, racial and ethnic, and socioeconomic differences [52], and various phenotypes that differ in symptoms spectrum (e.g. insomnia, fatigue) and occurrence and severity of hypersomnolence. Cluster analysis is crucial for a structured characterization of the phenotype, encompassing clinical presentation and pathophysiological concepts. In turn, such phenotyping is a prerequisite for risk stratification and personalized treatment [53,54].

The apnea-hypopnea index (AHI) is the most frequently used parameter of OSA severity and treatment indication despite poor correlation with hypersomnolence and inability to capture hypoxic burden, symptom severity, or other major outcome parameters. Given these shortcomings, the AHI should be at least used in conjunction with other biomarkers or measures of disease severity that may better capture symptom burden and need for treatment [55–58]. New biomarkers of OSA severity that have been proposed include the delta heart rate index [56], the sleep apnea hypoxic burden [59], and the pulse wave amplitude drops index [60]. Similarly, subjective and objective hypersomnolence estimates might differ [61,62]. Cardiovascular comorbidities with increased sympathetic activation may reduce hypersomnolence and promote other symptoms, such as fatigue or disturbed sleep. On the other hand, polypharmacy, age, sex/gender, ethnicity, and sociocultural factors including profession may favor overrepresentation of hypersomnolence.

A first-visit integration of subjective and objective evaluation of OSA symptomatology in an individual patient (including insomnia and fatigue scales) is paramount, without being limited to the Epworth Sleepiness Scale (ESS), which is not considered an exhaustive subjective estimate. The evaluation should include at a minimum a single question on the impact of the disease on the patient’s life (e.g., “Does sleepiness impact your daily life?”) as well as additional questions specifically to assess driving safety (i.e., “have you every fallen asleep while driving?” or “in the last few months have you experienced drowsy driving?”). This can be supplemented by additional validated questionnaires (Athens Insomnia Scale; Fatigue Severity Scale; Functional Outcomes of Sleep Questionnaire) and, in specific situations, by objective testing.

### 5.2. Management

Targeting hypersomnolence in OSA is of utmost clinical relevance, as there is growing evidence that it is associated with morbidity and mortality, although it is not clear whether hypersomnolence is a predictor or a causal factor to this extent.

The indication for OSA treatment is currently changing substantially. Over the last two decades, it was driven primarily by the number of breathing disturbances, as indexed by the AHI. Most recently, algorithms based on symptoms and comorbidities have been proposed [58, 63,64] towards personalized care and beyond the “one size fits all” decision process. Importantly, hypersomnolence has also been demonstrated as a marker of impaired cardiovascular outcomes [53,65]. Therefore, subjective and objective estimates of hypersomnolence represent a crucial step for risk stratification and initial therapeutic decision. The combination of hypersomnolence and other symptoms (such as insomnia) and cardiometabolic comorbidities and depression have greater impact on treatment decisions, while the number of breathing disturbances should be taken into account when exceeding prognostic relevant figures (e.g., AHI >30/h) [63]. Age, sex/gender, and sociocultural factors also influence therapeutic decisions and follow-up algorithms. Such multi-tier assessment should be applied to stratify patients at first visit and again at follow-up. There is a large consensus that lifestyle, continuous positive airway pressure (CPAP) therapy adherence, and the need for device or mask adaptation are paramount during follow-up, which also should actively involve the partner, if any [66]. However, the decisive prognosis predictors (hypersomnolence, comorbidities, residual hypoxia) and their longitudinal evolution should also play a central role in the re-assessment and decision-making during follow-up.

CPAP therapy usage correlates with an improvement in different clinical outcomes in a dose-response fashion. Therefore, it is preferable to recommend the longest device usage possible instead of a fixed threshold to cover the entire night, including major REM sleep episodes. Younger patients experience a more pronounced impact of CPAP treatment on sleepiness and quality of life compared to older patients [67]. In case of residual sleepiness, the diagnostic work-up should include treatment-associated problems, as well as medical, neurological (see section 6), and psychiatric (see section 7) diseases and other sleep disorders such as insufficient sleep syndrome. This re-evaluation step is also important for ruling out de novo sleepiness, developed after treatment initiation.

**Fig. 4.**
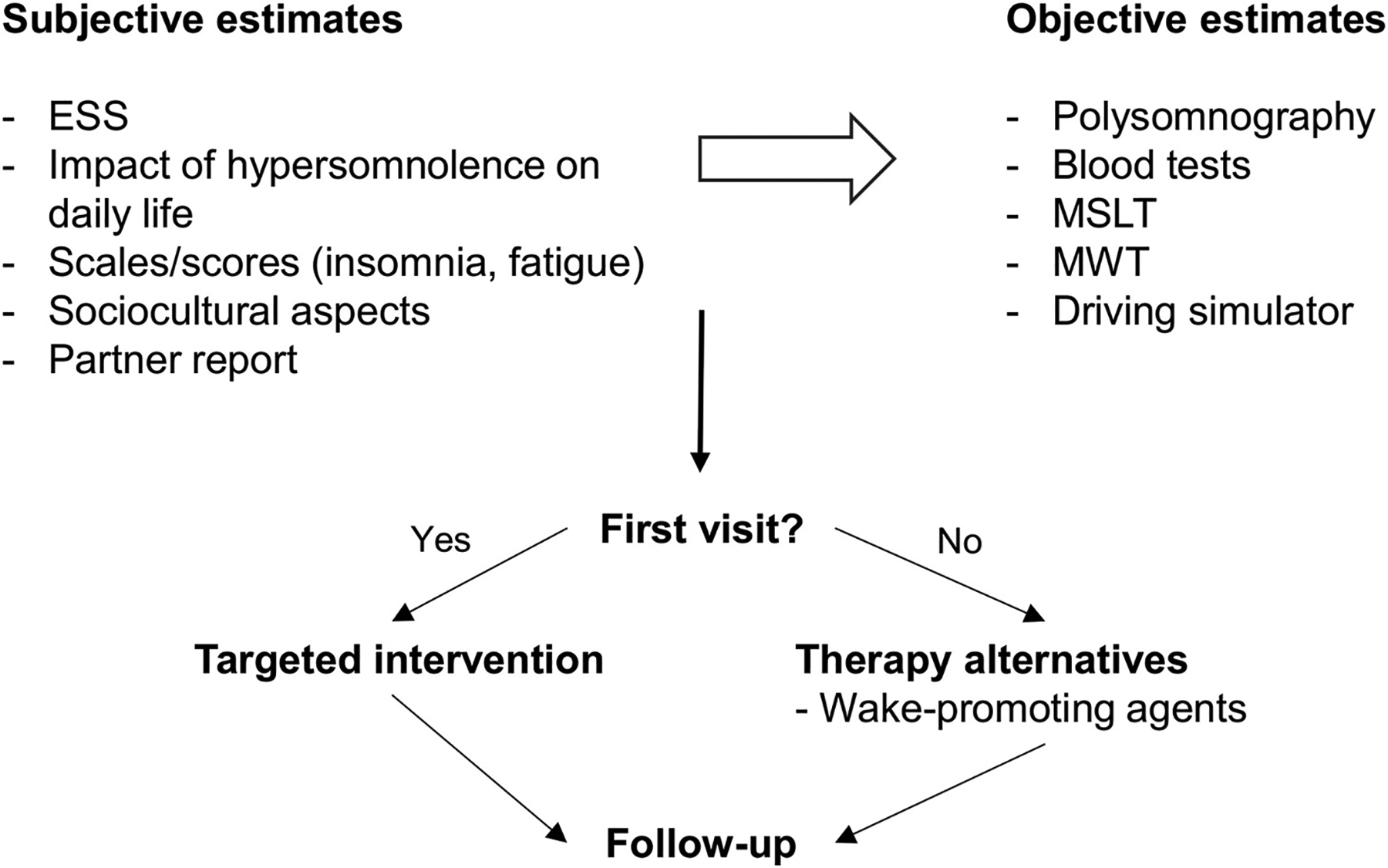
Summary of management of hypersomnolence in obstructive sleep apnea. ESS, Epworth Sleepiness Scale. MSLT, Multiple Sleep Latency Test; MWT, Maintenance of Wakefulness Test. Wake-promoting agents should be considered for administration only at follow-up (“First visit? No”), if necessary.

Should the patient report residual hypersomnolence despite optimal treatment, or not accept or tolerate CPAP therapy, wake-promoting agents (pitolisant, solriamfetol, modafinil) should be evaluated after a thorough discussion of benefits and side effects with the patient and evaluation of differential diagnoses, and after having repeated objective hypersomnolence estimates [61,68,69]. The first choice of pharmacological agents is driven by the associated cardiovascular risk [70]. The suggested strategy to manage hypersomnolence in patients with OSA is summarized in Fig. 4.

### 5.3. Research priorities

There is an urgent need for identifying biomarkers of OSA severity and treatment indication that complement the AHI, including in the correlation with hypersomnolence. A more differentiated analysis of patients’ subgroups with OSA is required, as study cohorts of patients with OSA do not always represent the usual OSA cohorts seen in clinical routine [71]. The understanding of the importance of subjective vs. objective hypersomnolence in guiding OSA patient stratification and treatment strategies also needs to be improved. In this respect, prospective clinical studies are warranted to clarify whether hypersomnolence is a predictor or a causal factor of morbidity and mortality in OSA. Further prospective studies are also required to determine clusters of hypersomnolence, comorbidities, hypoxic burden, OSA-induced autonomic activations and socioeconomic factors, and for validating an integrative approach. Important research questions may also be assessed by studies on OSA with other designs, as each design shows specific advantages and limitations [72]. Moreover, the role of hypersomnolence as a predictor of treatment response to CPAP therapy requires further investigation. Further evidence on the role of CPAP device usage patterns instead of crude usage duration is required. Finally, head-to-head comparison between wake-promoting medications and stimulants in randomized controlled trials is also required in this population.

## 6. Hypersomnolence in neurological disorders

### 6.1. Epidemiology and phenotypes

Hypersomnolence is one of the most frequent symptoms in neurological diseases [73,74] (Table 3). EDS is observed in 20–50 % of central nervous system disorders, depending on the definitions (see section 3) and means of assessment [75]. Data on ENS, however, are very limited. For peripheral neurological disorders, almost no epidemiological data on hypersomnolence are available. Data have only been published for individual diseases, such as for Charcot–Marie–Tooth disease: in a series of 257 patients, 23 % reported EDS, as assessed by ESS scores >10 [76].

EDS is frequently found in neurodegenerative disorders as well as in vascular and inflammatory neurological disorders. In inflammatory disorders, fatigue is also common and its differentiation from hypersomnolence may sometimes be difficult [77]. The frequent comorbidity of hypersomnolence is probably linked to the neurobiology of sleep and neurological functions (see section 4).

There is a bidirectional relationship between hypersomnolence and many neurological disorders. For instance, hypersomnolence is a risk factor for stroke [78], but can be also a resulting symptom after stroke [79,80]. Furthermore, hypersomnolence in neurological disorders may be associated with cognitive decline [81], depression, risk of accidents, and reduced quality of life [82]. In many neurological disorders, fatigue [83] or apathy (e.g., in Parkinson’s disease) is often present [84–88] (Table 3). Different phenotypes, such as EDS, ENS and fatigue, can present together or in variable combinations. However, one phenotype is often dominant or at least accentuated. For instance, EDS is the predominant phenotype in Parkinson’s disease [84–88], whereas fatigue is frequently present in multiple sclerosis [89–91] and may occur after stroke [79].

EDS and ENS may reflect different pathophysiological mechanisms, such as increased sleep drive and insufficient arousal (see section 4). Only in rare cases can the symptom be attributed to specific anatomical lesions (e.g. thalamic stroke lesions causing ENS) [92], or physiological disturbances (i.e. orexin deficiency in narcolepsy) [93].

The subjective perception of EDS or ENS and their severity and significance may vary from objective findings. However, subjective and objectives measures of hypersomnolence are often congruent in neurological disorders, in contrast to OSA (see section 5) and psychiatric disorders (see section 7). In most neurological disorders, EDS is chronic and remains stable over time. In some cases, EDS intensity fluctuates over time, or a progression of EDS occurs [94]. Although they are frequently found in neurological disorders with hypersomnolence, OSA or periodic limb movements during sleep (PLMS) may not contribute significantly to EDS in neurological disorders, as suggested by the frequent lack of relevant improvements of hypersomnolence after OSA or PLMS treatment [95].

### 6.2. Management

The management of hypersomnolence in neurological disorders is complex due to its multifactorial genesis (see section 4), which may stem from: (i) sleep impairment, including qualitative and quantitative sleep deficits and fragmented sleep; (ii) pathological abnormalities of the central nervous system with alterations in arousal and/or sleep regulatory circuits; (iii) misalignment between the circadian rhythm and the sleep/wake cycle; (iv) drugs, and (v) comorbidity with psychiatric or other primary sleep disorders. However, management of hypersomnolence is critical for its impact on quality of life and increased risk of injuries/accidents.

The management of hypersomnolence in neurological disorders consists of a combination of facilitating awareness/acceptance of the neurological disorder, behavior and lifestyle advice including light exposure, nutrition, and physical activity, and pharmacologic interventions. In general, patients should be advised to have a consistent schedule, go to bed at the same time each night, and get up at the same time each morning as much as possible. In narcolepsy, according to guidelines, scheduled daytime naps, usually less than 20 min, may temporarily alleviate and prevent daytime sleepiness, and a short nap just before certain activities demanding a high degree of attention may facilitate the proper execution [96]. The optimal frequency and duration of naps must be established on an individual basis. However, long naps may have no refreshing properties due to subsequent sleep inertia [97]. Indeed, in idiopathic hypersomnia, many patients prefer avoiding naps because of the post-napping inertia. Unfortunately, there are few published studies regarding this topic. In idiopathic hypersomnia, adapted work schedules allowing patients to sleep longer and arrive later in the morning may be useful. Teleworking and flexible working hours may be beneficial in narcolepsy and idiopathic hypersomnia and should be applied whenever possible.

Dopaminergic therapies, including levodopa and dopaminergic agonists, can cause or aggravate EDS. Several studies indicated that EDS occurs more frequently in patients on pramipexole than those on placebo or other drugs to treat Parkinson’s disease [98]. Notably, the risk of daytime sleepiness is related to the dose of dopaminergic medications. EDS is more common in patients with Parkinson’s disease taking higher dosages of dopaminergic agonists and levodopa, typically in the dose-increasing phase or with time on a stable dosage. Divergent affinity for D1 and D2 dopaminergic receptors could result in different effects of dopaminergic drugs on EDS. Pramipexole and ropinirole are mainly D2 dopaminergic receptor agonists. Striato-nigral neurons expressing D1 dopaminergic receptors have been demonstrated to promote wakefulness whereas striato-pallidal neurons expressing D2 dopaminergic and A2a adenosinergic receptors induce sleep [99]. Clonazepam used to treat REM sleep behavior disorder may also be a possible iatrogenic cause of EDS in Parkinson’s disease, due to its long half-life.

There are guidelines of European [96] and American [100] scientific societies for pharmacological treatment of hypersomnolence in neurological disorders. The main recommendations of a task force of the European Academy of Neurology, European Sleep Research Society and European Narcolepsy Network for the management of EDS in narcolepsy [96] are the following: for adults, scheduled naps, modafinil, pitolisant, sodium oxybate, solriamfetol (all strong), methylphenidate, and amphetamine derivates (both weak); for children, scheduled naps, sodium oxybate (both strong), modafinil, methylphenidate, pitolisant, amphetamine derivates (all weak). Recently, recommendations for children have been slightly revised [101]. It should be considered that measures of specified outcomes such as EDS varied substantially from study to study and consisted of both subjective assessments (ESS and Clinical Global Impressions Scale) and objective measures (MSLT and MWT). As previously mentioned, subjective and objective measures do not always correlate consistently, although they are more likely to do so in neurological disorders. Several studies included the MSLT as a primary objective outcome measure. However, as discussed in section 3, the MSLT has limitations, and while it remains important for diagnosis, it is less relevant as an outcome measure. Moreover, several studies included participants with narcolepsy type 1 and type 2 but did not separate effects between these two disorders. Amongst patients there is considerable inter-individual variation in the efficacy and side effect profile of commonly used drugs. Therefore, there is a need for a precise individual treatment plan according to symptom pattern, disease severity, and comorbid conditions.

In the guidelines of the American Academy of Sleep Medicine for the treatment of central disorders of hypersomnolence [100], the authors made the following recommendations: in idiopathic hypersomnia, to use modafinil (strong), or clarithromycin or methylphenidate or pitolisant or sodium oxybate (all conditional); in Kleine-Levin syndrome, to use lithium (conditional); in dementia with Lewy bodies, to use armodafinil (conditional); in Parkinson’s disease, to use modafinil or sodium oxybate (both conditional); in posttraumatic hypersomnia, to use armodafinil or modafinil (both conditional); in myotonic dystrophy in adults, to use modafinil (conditional); in multiple sclerosis in adults, to use modafinil (conditional).

### 6.3. Research priorities

Overall, there is a need for better identification of specific outcome measures for hypersomnolence in neurological disorders, and better definition of how to measure severity, who requires a drug intervention, how to treat, and how to assess improvements. This includes a better understanding of the impact of hypersomnolence on the course and prognosis of neurological disorders, also incorporating patient-reported outcomes. Additional and especially objective data on ENS are needed, and peripheral neurological disorders should also be systematically evaluated for hypersomnolence. Future studies should consider sociodemographic, ethnic, gender, language and cultural factors as well as lifestyle issues such as diet and exercise, which may have an impact on hypersomnolence. Pharmacological trials should include more head-to-head drug comparisons along with studies that use drug combinations and evaluate long-term compliance. More clinical trials with orexinergic compounds for hypersomnolence in neurological disorders are particularly needed, in light of the promising results obtained with orexin receptor 2 selective agonists on patients with narcolepsy type 1 [102].

## 7. Hypersomnolence in psychiatric disorders

### 7.1. Epidemiology and phenotypes

Hypersomnolence is a very common complaint in psychiatric disorders, particularly mood disorders. Cross-sectional prevalence varies widely depending on the definition for hypersomnolence (see section 3), study methodology, and disorder in question, with estimates ranging between 5 % and 78 % [103]. More recent estimates suggest hypersomnolence occurs in ~50 % of depressed persons referred for polysomnography [104]. In fact, the association between EDS and depression may be much stronger than that between EDS and OSA (effect sizes 10.6 vs. 1.2, respectively) [105] (see section 5). Some investigations have demonstrated increased proportions of hypersomnolence in women [106], while others have not demonstrated sex-related differences [107]. Most research has focused on adulthood, though some studies have suggested lower rates of hypersomnolence (8.9–21.7 %) among children with depression under 13 years, with substantial prevalence increases (26.9–56.8 %) in adolescents above this age threshold [103]. Notably, EDS has been demonstrated to be both a common residual symptom and severity marker of major depression in adolescents [108].

Very little is known about hypersomnolence in geriatric depression. Associations between daytime sleepiness and depressive symptoms among the elderly have been identified in community-based samples [109], however other studies examining sleep complaints within major depressive episodes have demonstrated approximately 3- to 4-fold reductions in the prevalence of hypersomnia in those above age 65 compared to young and middle-aged adults [110].

Despite variable but often high rates of hypersomnolence associated with mental illness, there is a paucity of data in this area compared to hypersomnolence related to OSA or neurological conditions, likely due in part to the rare application of sleep-specific questionnaires and measures in psychiatric research. In this vein, there are generally limited longitudinal data that have examined the role of EDS in the course of depression, likely because the majority of validated depression scales used in epidemiological studies do not assess EDS per se, and at best quantify excessive sleep duration akin to ENS. Still, data suggest that EDS and depression have longitudinal relationships with one another, suggesting a possible bidirectional relationship akin to what has been established between depression and insomnia [111–114].

Objective measurement of hypersomnolence in psychiatric disorders is complicated. The majority of persons with hypersomnolence occurring in the context of a psychiatric illness do not demonstrate abnormalities on the MSLT, with meta-analysis demonstrating a mean sleep latency of 10.9 min (95 % CI 9.4–12.4 min) [115]. Conversely, a sizeable minority of patients (~25–40 %) will demonstrate a mean sleep latency in the current pathological range [115,116]. Thus, delineating between psychiatric EDS and other disorders in the narcoleptic borderland, particularly idiopathic hypersomnia, can be challenging. It is an open question whether a distinction should be made between idiopathic hypersomnia with comorbid depression and hypersomnia/hypersomnolence associated with a psychiatric disorder in the absence of known pathophysiology for either condition. For insomnia, no such distinction exists. Insomnia can occur without depression or can be comorbid with depression and vice versa. It is well established that insomnia and depression have a complex, bidirectional relationship. Both may have synergistic effects, and both should be treated as distinct but overlapping entities when present in an individual [114]. Regardless, the limited relationships between various objective and subjective measures of hypersomnolence, including among persons with depressive symptoms, highlight that hypersomnolence is multifaceted and may not be reliably captured by a single measure [117] (see section 3). This may explain some of the seemingly paradoxical relationships observed between objective sleep propensity measured by the MSLT and subjective EDS observed on both the patient level and in larger epidemiological studies [118]. Emerging data suggest that the psychomotor vigilance test may be a more salient objective measure of hypersomnolence in mood disorders, both cross-sectionally and longitudinally [119].

### 7.2. Management

The management of hypersomnolence in psychiatric disorders would benefit from accounting for the cause of the sleepiness, and tailoring treatment specifically to each individual [120]. The heterogeneity of sleep symptoms and phenotypes that occur in psychiatric disorders complicates research efforts and clinical care. EDS is often overlooked in psychiatric practice. Ideally, treatment of EDS should be directed towards the underlying cause, whenever possible. In the case of EDS occurring in psychiatric disorders, this requires a thorough evaluation for common medical and neurological disorders associated with EDS (see section 6), as well as sleep disorders like OSA (see section 5), which is frequently co-morbid with depression [121]. Unfortunately, many in the behavioral health community are not skilled in the evaluation and differential diagnosis of EDS, nor mindful that many sleep disorders may present initially in psychiatric contexts. For example, patients with narcolepsy are often initially misdiagnosed and managed symptomatically by psychiatrists, which delays care and optimal treatment [122]. Further complicating the diagnosis of narcolepsy in persons with severe depression are recently published cases, however rare, of depressed persons without cataplexy and negative for HLA DQB1*0602, who were nonetheless orexin deficient [123]. In a larger retrospective series of orexin-deficient patients with narcolepsy type 1, seven patients (1.9 %) were found negative for HLA DQB1*0602 [124]. Thus, further educational efforts within the mental health community regarding sleep and its disorders are required, as well as further research on the part of the sleep medicine community that assists mental health providers advance their assessment of EDS. There are a considerable number of unmet needs and long delays along the patient journey [125]; the importance of establishing an early diagnosis cannot be overemphasized.

Assuming no other disorder is responsible for EDS occurring with a psychiatric disorder, treatment itself is often symptomatic. There are no published controlled data of any agent that directly evaluate its effects on EDS in psychiatric hypersomnolence. Limited studies have either examined the effects of medications commonly used to treat psychiatric disorders on EDS in other illnesses or utilized secondary analyses of available data in psychiatric treatment studies. Each study design rarely uses objective measures of sleepiness and often relies on suboptimal subjective tools to quantify EDS. The literature in this area is so scant that the American Academy of Sleep Medicine in its most recent treatment guidelines gave no recommendations for specific interventions in the treatment of hypersomnolence associated with a psychiatric disorder [100]. Thus, at present, the treatment of EDS in psychiatric disorders remains more an art than a science, with practitioners often using many of the same wake-promoting agents used to treat other central nervous system disorders of hypersomnolence, as well as alerting antidepressants that broadly support components of the ascending arousal mechanism (e.g. dopaminergic ventral periaqueductal gray, noradrenergic locus coeruleus). Clinical trials of different compounds are required to develop an evidence base on which clinical practice in this domain can advance.

### 7.3. Research priorities

Beyond building an evidence base for treatment of hypersomnolence in psychiatric disorders, enhanced understanding of the biological underpinnings of EDS/ENS in these illnesses will be paramount to advance the field. This is particularly challenging since, unlike the afore discussed OSA and neurological disorders, the pathophysiology of psychiatric conditions themselves are not known. The causes of EDS in psychiatric disorders are almost assuredly multifactorial (see section 4), and there is evidence for both circadian misalignment and sleep homeostatic function being involved [126,127]. Other potentially fruitful areas of inquiry may include clarification of the role of orexin in psychiatric disorders [128], as well as non-pharmacological (e.g. cognitive behavioral therapy for hypersomnolence [129]) and somatic therapies (e.g. transcranial magnetic stimulation [130]) on psychiatric EDS. Importantly, evaluating the effects of targeted EDS treatment on other psychiatric symptoms may help further elucidate their complex, and likely bidirectional, relationships.

## 8. Impact of hypersomnolence on daily activities, work productivity and quality of life

As prevalent as hypersomnolence is, there is still a dearth of knowledge regarding its societal and individual costs. Nevertheless, hypersomnolence has been associated with considerably higher health-related costs with lower levels of employment and income [131]. The annual mean excess health-related cost of hypersomnolence was calculated at €3,498 for patients and €3,851 for their partners. A systematic review on OSA estimated annual costs across Europe to range between €1,669 and €5,186, with indirect costs comprising 25–51 % of the total costs [132]. Evidence linking OSA severity to costs was considered inconclusive [132], and a review evaluating the economic and societal burden of EDS in patients with OSA found that only one of 38 publications assessed reported data for health-care utilization [133]. Nevertheless, EDS in OSA has been associated with higher healthcare utilization compared to patients without EDS [134,135]. In particular, a cross-sectional analysis showed significantly higher outpatient visits among patients with OSA and residual EDS compared to OSA patients without EDS and increased risk of hospitalizations (odds ratio 3.9, 95 % confidence interval 1.0–12.0, p = 0.046) [135].

Another analysis comparing patients with narcolepsy to matched controls reported two to three times more hospitalizations, emergency room and outpatient visits among patients with narcolepsy over a 6-month time span (all p < 0.0001) and significantly higher costs associated with the aforementioned work absenteeism ($12,839 vs. 7,621), hospitalizations ($27,642 vs. $10,998), and outpatient visits ($22,828 vs. $12,667) (all p < 0.001). Additionally, narcolepsy patients reported 70 % more long-term disability (p = 0.004) [136].

The direct costs associated with informal care remain underexplored. Studies in Australia estimated that these costs contribute approximately 1 % of the total costs for inadequate sleep, mostly with EDS [137], and of moderate to severe OSA with EDS [138].

The Institute for Health Metrics and Evaluation (www.healthdata.org) continuously evaluates the global burden of disease, including 371 diseases world-wide. Unfortunately, sleep disorders are not separately included in this evaluation. Lubetkin et al. [139] evaluated the effect of sleep duration on health-related quality of life and quality-adjusted life years of persons aged >65 (n = 2380). The quality-adjusted life years of participants who reported between 7 and 9 h of sleep per night were significantly higher than those of participants who slept ≥10 h/night (17.6 vs. 7.8). Health-related quality of life (EQ-5D) was lower, and mortality rates were higher for participants who reported feeling unrested during the day. In fact, health-related quality of life (SF-36 score) for idiopathic hypersomnia is reduced in most domains. Even patients receiving drug treatment exhibit lower scores than national normative data [140]. Furthermore, EDS naturally affects one’s ability and willingness to engage in both work and personal daily activities [42]. This may be especially important for adolescents whose academic performance [141] and social connectedness [142,143] are impaired, setting the stage for their future health-related quality-of-life trajectory.

### 8.1. Research priorities

It is difficult to calculate the societal and individual costs of hypersomnolence, partially due to the high prevalence of comorbidities. One option would be to focus on medication use rather than diagnosis as an indicator of disease prevalence. National databases for income, such as those in Scandinavia, could then potentially be utilized to estimate costs. Furthermore, the impact of treatment could be evaluated using claims data in, e.g., France and the US. Further research should address cost components across age groups. In minors, poor sleep negatively impacts brain development and academic performance, potentially leading to long-term financial disadvantages [144]. This would also create a possibility for influencing the political community; valuable data could have an impact on future treatment policies to help more people. Sleep education may also help in some instances [145], but more research is needed to elucidate which aspects of such education would be fruitful.

## 9. Conclusions

The primary take-home message that emerged from the Task Force discussions is that hypersomnolence is both prevalent and heterogeneous in its manifestations in a wide variety of pathological conditions encompassing OSA and neurological and psychiatric disorders. The Task Force supported the recent attempt to revise definitions of terms related to hypersomnolence [1,146] as a step forward in clarifying phenotypic heterogeneity and making such heterogeneity explicit. Recent findings, building upon decades of research, highlight multiple pathophysiological pathways potentially involved in hypersomnolence. However, knowledge of the specific causal factors for hypersomnolence remains the exception rather than the rule, and the specific factors responsible for EDS vs. ENS and of subjective vs. objective disorders remain largely unclear. The uncertainty regarding which factors are mostly responsible for hypersomnolence in individual patients is an important limitation to the development of personalized approaches to diagnosis, prognosis, and management of hypersomnolence. In the pursuit of biomarkers for hypersomnolence, ethical considerations should play a crucial role, with significant privacy concerns that should be duly reflected in the documents and procedures to obtain informed consent from the study participants. For instance, the identification of a genetic marker linked to susceptibility to sleepiness could significantly affect an individual’s fitness to drive or impact their insurance profile. These considerations highlight the importance of safeguarding both privacy and the ethical management of sensitive information in research particularly in this field.

Although limited, the available evidence indicates that hypersomnolence entails substantial costs, both economically and personally, and substantially impacts quality of life. This further emphasizes the urgent need to improve hypersomnolence diagnosis and management, leveraging the different perspectives that may be gained on hypersomnolence with discussion across multiple clinical fields.

## Figures and Tables

**Table 1:** Proposed definitions of terms pertaining to hypersomnolence.

	Excessive daytime sleepiness (EDS)	Excessive need for sleep (ENS)
Clinical symptoms/complaints	[EDS/ENS] Feeling of sleepiness (as opposed to fatigue) for most of the day.	
	[EDS1] Inability to stay awake in monotonous situations with unintended napping and possible sleep attacks.	[ENS1] An acquired increased need for sleep in normal daily life, including ≥10 h of sleep per 24 h and/or ≥ 9 h of nocturnal sleep.
	[EDS2] Acquired need for scheduled napping during the day.	[ENS2] In case of only minor complaints of sleepiness (i.e., [EDS/ENS]), daily occurrence of sleep inertia/sleep drunkenness.
	[EDS3] Difficulty with sustained attention and vigilance.	[ENS3] Sleep extension will not significantly improve the daytime complaints (i.e., [EDS/ENS] and [ENS2]).
	[EDS4] Automatic behaviours that can be attributed to EDS.
Diagnostic criteria	Daily or near daily occurrence of [EDS1] OR daily occurrence of [EDS/ENS] and ≥1 of the other symptoms listed (i.e., [EDS2–4]).	Daily or near daily occurrence of [EDS/ENS] AND [ENS1] AND [ENS2] AND [ENS3].

Definitions are adapted from [1].

**Table 2:** Differences between Mean Sleep Latency Test and Maintenance of Wakefulness test.

	MSLT	MWT
Measure of sleep resistance		*
Measure of sleep propensity	*	
Pathological values	≤8 min	≤19 min
Measure of REM sleep intrusion	*	
PSG night before	Mandatory	Recommended
Sensitivity to sleep deprivation	**	**
Fitness to drive		**
Diagnosis of narcolepsy	*	
Treatment response (drugs, behavior, CPAP)		**
Correlation with ESS	Weak to moderate	Weak to moderate
Correlation with reaction tests	Weak to moderate	Moderate to strong

The number of stars indicates the appropriateness of test for each of the items. MSLT, multiple sleep latency test. MWT, maintenance of wakefulness test. REM, rapid-eye-movement. PSG, polysomnography. CPAP, continuous positive airway pressure. ESS, excessive daytime sleepiness.

**Table 3:** Hypersomnolence in neurological disorders.

Neurological Disorder	Hypersomnolence	Fatigue	Remarks	References
Parkinson’s disease	20–40 %	30–70 %	Apathy in 10 %	[84–88]
Alzheimer disease	20–50 %			[147]
Stroke	20–40 %	40–60 %	Often ENS in thalamic/sub-cortical stroke.	[78–80]
Epilepsy	20–30 %			[148,149]
Multiple Sclerosis	10–20 %	>70 %	Rarely symptomatic narcolepsy	[89,90,150]
Neuromuscular disorders	20–40 %	40–60 %		[151,152]
Autoimmune/paraneoplastic	28 %	61–65 %		[153,154]
Migraine/headache	20–40 %			[155]
Narcolepsy	>95 %	>70 %		[156,157]
Idiopathic hypersomnia	>95 %	>70 %		[2]
RLS	10–20 %	20–40 %		[158]

RLS: restless legs syndrome; ENS: excessive need for sleep.

## BOX 1 Hypersomnolence in children and teenagers

- Children require more sleep (10–14 h per day).
- Napping is common.
- Possible causes of excessive daytime sleepiness (EDS) are inadequate sleep duration, sleep apnea, inconsistent sleep routines, growing pains, developmental changes, and school-related stress.
- Children and teenagers with developmental disorders (e.g., attention-deficit/hyperactivity disorder) are at higher risk for EDS due to irregular sleep patterns.
- Identification of EDS is more difficult than in adults and often relies on parents or caregivers.
- EDS often manifests through irritability, difficulty concentrating, hyperactivity (as a compensatory mechanism), or emotional outbursts.
- Sleep-deprived children and adolescents may have difficulty interacting socially and may show signs of anxiety or mood swings.
- EDS can negatively impact school performance.
- Early recognition of EDS is even more important than in adults.
